# An accessible and open-source workflow for fabrication of custom PDMS culture chambers using 3D printing

**DOI:** 10.1016/j.mex.2026.104091

**Published:** 2026-08-06

**Authors:** Antonio Algarín, Paula Lian Cabrera, Santiago F. Scagliusi, Paula Daza, Alberto Yúfera, Daniel Martín

**Affiliations:** a Departamento de Tecnología Electrónica, ETSII, Universidad de Sevilla, Spain; b Instituto de Microelectrónica de Sevilla (IMSE), Consejo Superior de Investigaciones Científicas/Universidad de Sevilla, Spain; c Departamento de Biología Celular, Facultad de Biología, Universidad de Sevilla, Spain

**Keywords:** Tissue engineering, Parametric design, Additive manufacturing

## Abstract

Custom cell culture platforms are widely used in tissue engineering applications. However, their fabrication often requires specialized microfabrication equipment and advanced technical skills, limiting accessibility. This work presents an accessible and open-source workflow for the fabrication of custom PDMS culture chambers. This workflow focuses on the use of parametric CAD designs, 3D-printed molds, resin casting and plasma-free bonding. The proposed approach allows users to generate customized molds adapted to the experimental requirements using a graphical interface without extensive CAD knowledge. The molds can be manufactured using commonly available 3D printers or outsourced at relatively low cost. Device assembly is performed using uncured PDMS as a bonding agent, eliminating the need for plasma bonding equipment. The resulting culture chambers were validated through watertightness testing and biological evaluation using N2a cell cultures. Cell viability after 72 h of culture was comparable to that observed in standard culture plates. By reducing requirements for specialized equipment and technical expertise, this workflow facilitates the rapid, low-cost and reproducible fabrication of customized PDMS culture platforms for tissue engineering and related biomedical applications.

## Graphical abstract

**Figure:**
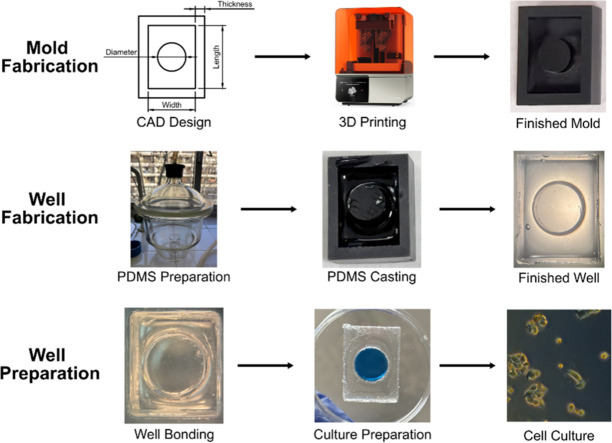

**Specifications table**

**Table:** 

**Subject area**	Engineering
**More specific subject area**	Tissue engineering
**Name of your method**	Manufacturing of PDMS culture chambers
**Name and reference of original method**	None
**Resource availability**	https://github.com/danmarfer/PDMS-Wells-OpenSCAD-Designs

## Background

The recent trends in biomedical and biological engineering have established a strong relation between tissue engineering and material science, with promising fields like electrical stimulation or microfluidics as an example [1]. This creates a highly multidisciplinary paradigm in which a vast array of knowledge and skills is needed: material science, design, biology, etc. To conduct research in these fields, a team has to have an assortment of profiles and a lab that is equipped for very diverse techniques. As increasingly specialized equipment and technical expertise are required, research accessibility becomes more limited. This problem is particularly common in tissue engineering, in which the use of custom cell culture platforms is usually unavoidable, creating the need to design and manufacture customized biocompatible cell culture platforms tailored to specific experimental requirements [2]. Unlike commercial well plates, these platforms can be integrated with custom hardware and fabricated in geometries that match the experimental design. Many platforms require customized culture chambers that can be integrated with electrodes, biosensors, microfluidic channels or other specialized substrates. Likewise, researchers often need culture wells with non-standard geometries or dimensions that match a specific device or experimental setup rather than the fixed formats provided by commercial cultureware. Examples include electrical stimulation platforms, organ-on-chip and microfluidic devices, biosensing platforms, and custom imaging or electrophysiological assays. The most common base material for these chambers is Polydimethylsiloxane (PDMS). PDMS is a silicone-based elastomeric polymer widely used in biomedical engineering. It is the standard for microfluidic devices as it is very easy to use, has favorable mechanical, optical and biocompatibility properties [3]. The fabrication of PDMS microfluidic devices generally relies on microfabrication techniques such as soft lithography [4]. For simpler devices, however, PDMS can be cast in molds produced by additive manufacturing techniques [5], reducing fabrication complexity. Nevertheless, the assembly of PDMS devices often still relies on plasma treatment to bond PDMS to glass or polymer substrates [6]. Plasma bonding requires specialized equipment that may not be available in many biological laboratories, representing an additional barrier. Therefore, there remains a need for workflows that minimize dependence on specialized equipment while maintaining customization. Although 3D printing is an accessible technique, computer aided design (CAD) modelling of the device is necessary and extensive design knowledge can be required even for the simplest parts, as designing molds is not trivial. The use of open-source parametric CAD models can help overcome this limitation, as such models can be modified to the required dimensions without extensive CAD expertise. This approach is common within the 3D printing community, where users often need to customize existing designs rather than create new ones from scratch. One of the most widely adopted solutions is OpenSCAD, an open-source software package for creating solid 3D models through a script-based workflow. In OpenSCAD, device dimensions and features can be defined as parameters, allowing users to generate customized designs by modifying a them instead of redesigning the model. This reduces the technical barrier while preserving the flexibility required for custom devices. Additionally, the text-based nature of OpenSCAD facilitates sharing, reproducibility, and version control. It also makes it compatible with modern AI-assisted coding tools.

In this work, we present an accessible workflow for the fabrication of custom PDMS cell-culture devices that combines parametric OpenSCAD mold design, mold fabrication by 3D printing, and device assembly using uncured PDMS as a bonding agent, eliminating the need for plasma treatment. By reducing the requirements for CAD expertise, microfabrication infrastructure, and specialized bonding equipment, the proposed method facilitates the rapid fabrication of customized and low-cost PDMS devices in laboratories with limited engineering resources.

### Method details

The protocol consists of three major stages: mold fabrication, PDMS well fabrication, and well preparation for cell culture. Firstly, the design is modified using the graphical interface of OpenSCAD to fit the dimensions needed. The mold is then 3D printed. PDMS is prepared and degassed, poured in the mold and let to cure for 48 h at room temperature (RT). After demolding the part, the well is bonded to the culture surface using uncured PDMS. The last step is to prepare the well for cell culture by ultraviolet (UV) sterilization.

### CAD designand mold fabrication

**Fig. 1:**
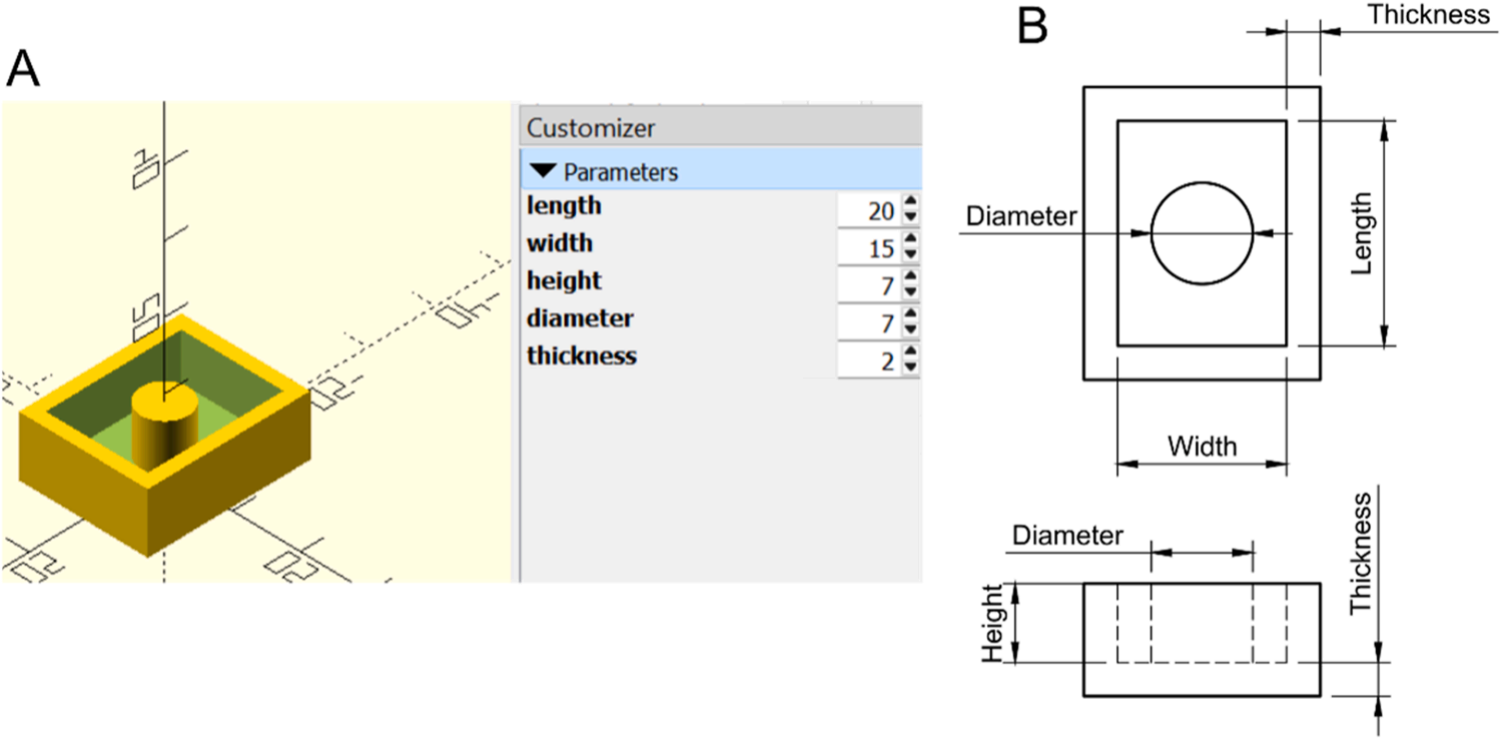
(A) OpenSCAD screenshot showing the “customizer” window. (B) Blueprint of the mold with the design parameters.

The molds have been designed to produce rectangular wells with a centered circle opening. OpenSCAD has a user interface called “Customizer” that lets the user modify the parameters of the design without the need to understand the code (Fig. 1A). The parameters defining the model refer to the resulting well, thus simplifying the task for the user. The length, width, height and diameter of the opening are the key dimensions. The last parameter refers to the thickness of the mold walls. All of the parameters are represented alongside the mold design in Fig. 1B

**Fig. 2:**
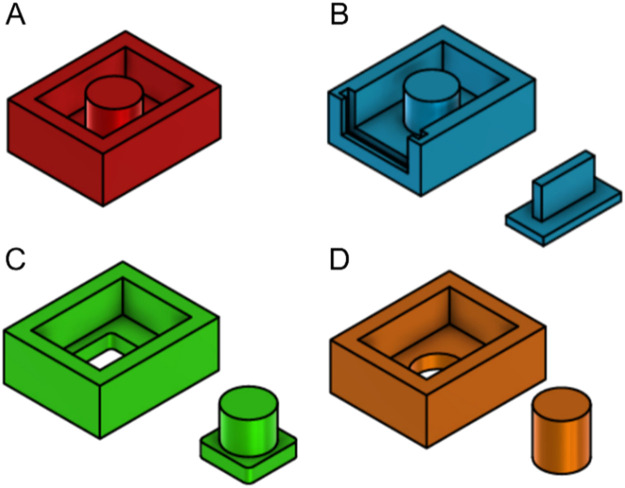
CAD Models of the 4 molds: (A) one-part mold, (b) two-part mold with gate, (C) two-part mold with removable base and (D) two-part mold with removable cylinder.

Four different designs were made (Fig. 2). The simplest is a one-part mold, as the removal of the part can be difficult, three two-part molds are also available. These designs have a removable part: a gate, the base or the cylinder that forms the opening. These molds facilitate the removal of the part but are more complex. Some designs have unique parameters that are self-explanatory.

After finishing the modifications on the CAD design, an STL file can be exported directly from OpenSCAD. Since 3D printing is dependent on the equipment and a well-known technique with a variety of tutorials and procedures available online [7,8], the process will not be explained in detail here. That said, a couple of considerations need to be made: firstly, the mold has to be printed flat, even in resin printers, this is so that the base of the mold has the least layer lines. Secondly, when using resin printers, which is recommended due to their higher overall resolution, the UV-curing step should be performed thoroughly, as traces of uncured resin can interfere with the curing process of PDMS, rendering the well non-biocompatible. For that matter, it is recommended to cure the parts 24 h at 60 °C under UV light. Lastly, if using filament printers, the bigger layer lines could result in wells that are not fully transparent. To address this, we recommend the use of ABS filament and doing mild acetone vapor smoothing of the mold. This, if done mildly, will smooth the layer lines with minimum change in the dimensions of the mold [1,9]. Plenty of resources are available online on this matter. Finally, if a 3D printer is not available, the design can be manufactured by external companies for a relatively low cost, OpenSCAD has a function that uploads the design to a selection of manufacturing companies directly. This allows for easy, accessible and affordable mold manufacturing, even when a 3D printer is not available.

### PDMS well fabrication

**Fig. 3:**
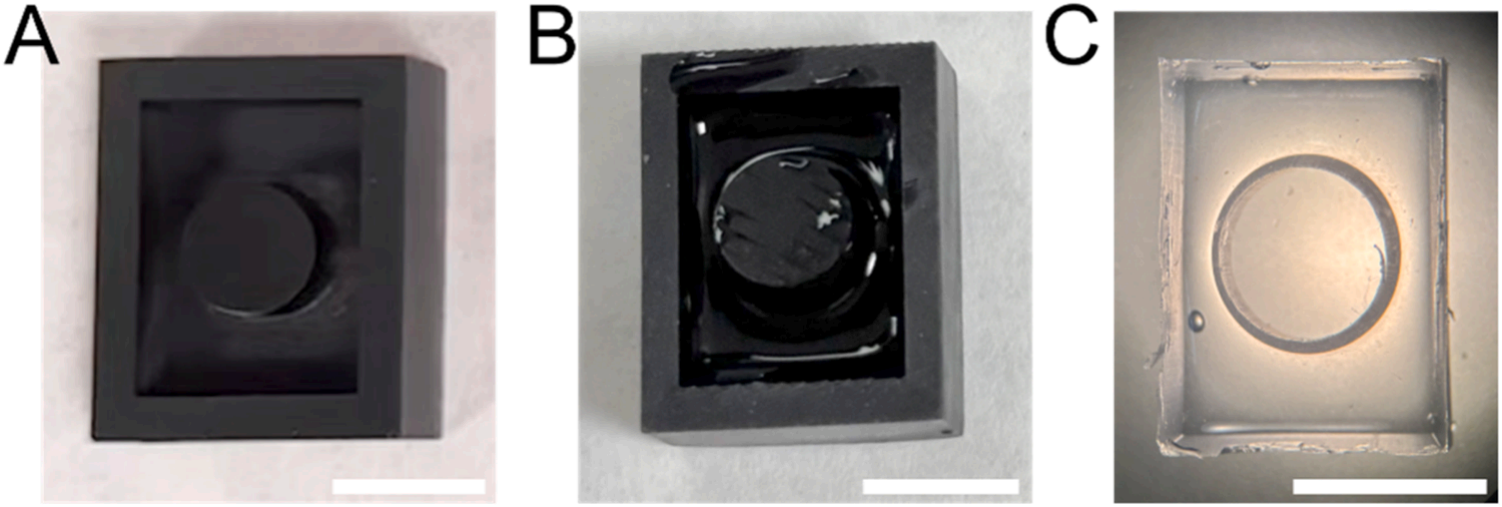
(A) Resin printed mold, (B) mold filled with PDMS and (C) PDMS well. Scale bar represents 10 mm.

The PDMS used was the Sylgard 184 from Dow. It is formulated as a two-part resin with a base and a curing agent that have to be thoroughly mixed at a weight ratio of 1:10. After mixing, a considerable number of air bubbles will be present that have to be degassed, preferably in a vacuum chamber. If such equipment is not available, the bubbles will eventually rise to the surface at atmospheric pressure if sufficient time is allowed, after which they can be popped with a sharp object like a needle. After degassing, the resin can be poured into the mold, there is no need to treat the molds with any demolding agent, however, the demolding process can be laborious especially for the one-part mold. Due to the high viscosity of the PDMS resin, it is recommended to use a small beaker to fill the mold or a Pasteur pipette with the tip cut; in case of a very small well, a needle can be used. If the two parts of the mold fit tightly, no resin should leak into the center hole. PDMS can be cured for 2 h at 70 °C, 4 h at 60 °C or 48 h at 25 °C. In our experience, curing for 24 h at 28 °C produced satisfactory results. PDMS does not swell or reduce while curing. After curing the part will be ready for demolding. For that, the use of a lancet or a scalpel can make the task easier. The different stages described in this paragraph are represented in Fig. 3.

### Well bonding and preparation for culture

**Fig. 4:**
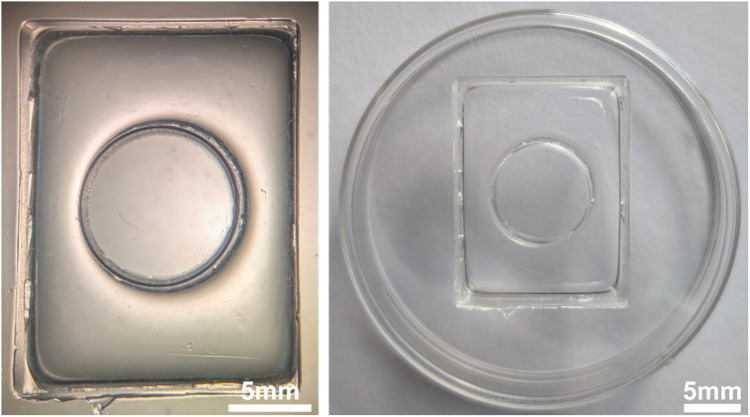
PDMS well after bonding.

The PDMS well is transparent and biocompatible as long as the curing process has been carried out successfully. If the part displays a sticky surface the PDMS curing process has failed, this can happen if the mold is not fully cured. The wells are durable and do not require overly cautious manipulation. To prepare the wells for culture, they are washed with soap, after which they are dipped in 70% ethanol for a minute. The usual procedure in microfluidics is to bond PDMS parts to glass using plasma treatment, nevertheless, using uncured PDMS as a bonding agent was found to yield very good results in glass and PET plastic surfaces. For that, another batch of PDMS is mixed and degassed, after which a thin layer of the resin is applied to the flat surface of the well using a lancet. The well is then gently pressed into the culture surface and cured again for 48 h at RT after which the well is fully bonded. No leakage of uncured PDMS beneath or into the culture chamber was observed when only a thin PDMS layer was applied. There is no need to clamp or apply weight to the part while curing. The PDMS layer should be thin enough to ensure no excess PDMS flows inside the opening. The bond created is strong enough to withstand the usual culture procedures. If necessary, this bond can be broken if significant force is used. The application of PDMS on the surface of the part improves transparency by reducing the imperfections in the surface of the piece. This bonding procedure can be avoided if the application does not require a very strong bond and both the part and the culture surface are very flat and clean; just pressing the PDMS well firmly against a flat surface will make it stick. An example of a well bonded to a petri dish using this procedure can be found in Fig. 4.

Wells can be bonded to standard culture surfaces like petri dishes, multiwell plates, glass slides, etc. or custom platforms like electrical stimulation or impedance spectroscopy electrodes. Once the part is bonded to the culture surface, the culture platform needs to be sterilized. For that the whole platform is dipped in 70% ethanol for 5 min inside a culture hood, after which it is exposed to UV light for one hour. Every side of the part needs to be exposed to UV for at least 30 min, in the case of wells with relatively small openings (<5 mm of diameter) it is recommended to tilt the part so that the bottom of the hole is not kept in the shadow and also to flip the part so every side of the well is exposed to UV light. After this the well is rinsed with sterile PBS and ready for culture.

### Method validation

**Fig. 5:**
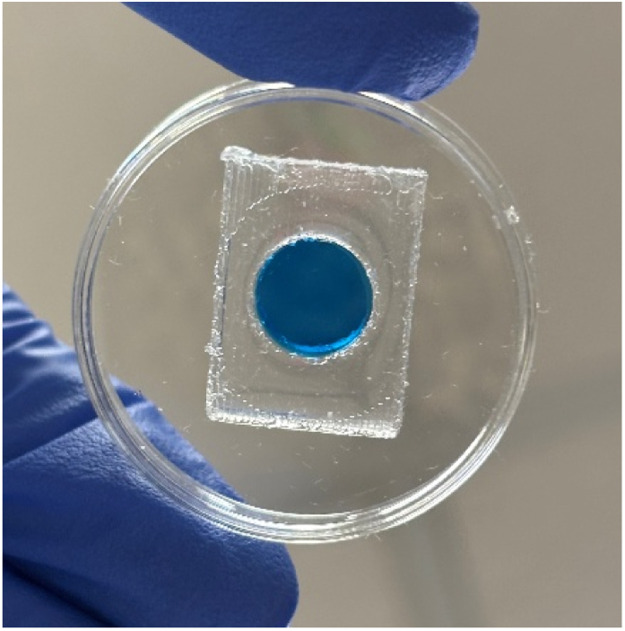
PDMS well watertightness test in 35 mm petri dish.

The wells were tested in two ways. Firstly, their suitability as culture platforms was assessed by their ability to retain liquid and remain watertight. Therefore, wells attached to 35 mm PET petri dishes were filled with distilled water dyed with methylene blue and left for 72 h. The liquid remained inside the wells without any spillage. The result of this test can be seen in Fig. 5.

**Fig. 6:**
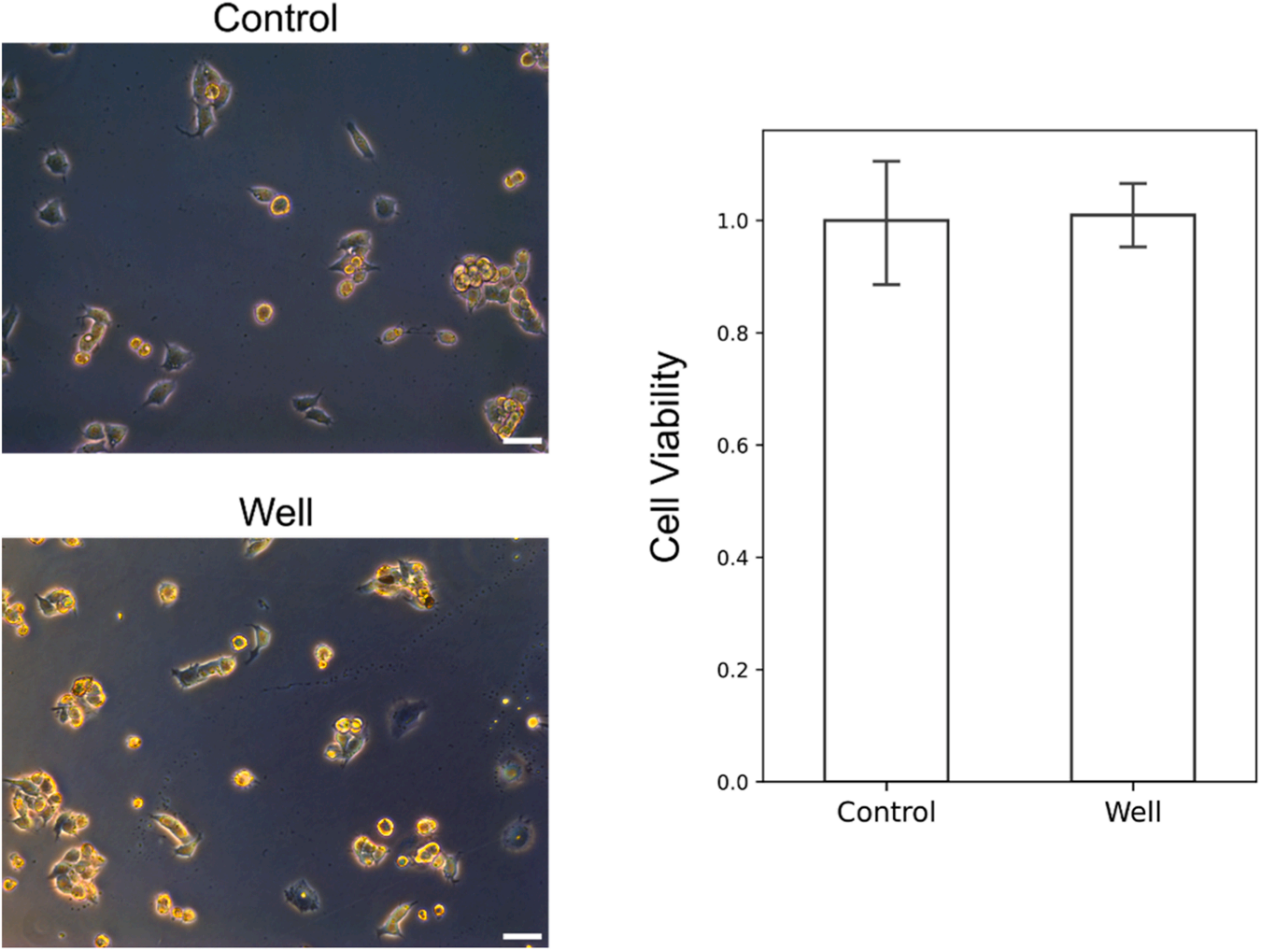
(Left) Representative images of N2a cells cultured for 24 h, taken at 20X, scale bar represents 20µm. (Right) Cell viability after 72 h as determined by sulforhodamine B assay (n = 2).

Secondly, and most importantly, the well biocompatibility was tested. For this, the viability of N2a cells cultured for 72 h in the well was assessed by sulforhodamine B assay, compared with a standard 96 well plate. The sulforhodhamine B assay is a colorimetric technique extensively used in cell biology to quantify cell density, as well as to determine viability and cytotoxicity in adherent cell cultures. The assay was carried as described in [2,10]. The number of cells seeded was adjusted to ensure equal cell density between conditions. 10,000 N2a cells were seeded in the PDMS well, while 5100 were seeded in the 96 well plate. This correction in cell number is due to the difference in surface area between the two platforms (almost double for the well). Fig. 6 displays representative images of cells cultured in the two conditions after 24 h and the viability results from the sulforhodamine B assay. We can see that cells in the well have a very similar density to the control. The morphology appears similar, however some cells in the well are rounder, most likely due to lower attachment. This slight difference could be a consequence of attachment issues or cell proliferation, as cells detach during mitosis. Sulforhodamine assay confirmed there is no difference in viability after 72 h of culture. This indicates that the wells do not affect the cells capability to proliferate, that said further experiments could help confirm the biocompatibility of the PDMS wells.

## Limitations

While the two-part molds help the demolding of the part greatly, it still takes some time and effort. The use of demolding agents could help in this case but may affect the curing process of the PDMS resin and thus the biocompatibility of the well. This has not been tested.

The CAD designs are limited to rectangular parts with a centered circle opening, different features, like squared openings for example, require the modification of the molds design. That said, modifying these designs should not be difficult for an experienced OpenSCAD user.

The method described forces the bonding of the PDMS well before the culture, meaning that cell seeding number has to be adjusted to the surface area of the well. Consequently, cells cannot be seeded in a conventional culture vessel and subsequently encapsulated inside these wells; the wells must be integrated into the culture platform before cell seeding.

## Ethics statements

Not applicable.

## CRediT author statement

**AA**: Conceptualization, Methodology, Writing - Review & Editing, Supervision. **PLC:** Validation, Investigation, Writing - Review & Editing. **SFC:** Investigation, Methodology, Writing - Review & Editing. **PD:** Methodology, Validation, Resources, Writing - Review & Editing. **AY:** Methodology, Writing - Review & Editing, Supervision, Funding acquisition. **DM:** Conceptualization, Methodology, Writing - Original Draft, Supervision.

## Supplementary material *and/or* additional information [OPTIONAL]

Not applicable.

## Related research article

None.

## Declaration of competing interest

The authors declare that they have no known competing financial interests or personal relationships that could have appeared to influence the work reported in this paper.

## Data Availability

Data is open-source. Available in the repository linked in the manuscript.
